# From Multi-Omics to Molecular Causality: A Five-Tier Evidence Framework for the Single-Bacterium–Metabolite Axis in IBD

**DOI:** 10.3390/microorganisms14081744

**Published:** 2026-08-08

**Authors:** Xingyue Song, Wanting Wang, Jingjing Yang, Xinning Liang, Wanli Ji

**Affiliations:** 1 School of Chemistry and Chemical Engineering, Shanghai University of Engineering Science, Shanghai 201620, China; xingyue_song@163.com; 2 Digital and Intelligent Empowerment Biomedical Innovation Center, School of Pharmacy, Shanghai University of Medicine and Health Sciences, Shanghai 201318, China; 13701840319@163.com (W.W.);

**Keywords:** inflammatory bowel disease, causality, microbiome, microbial metabolites, single-microbe–host interaction

## Abstract

Gut microbiota research in inflammatory bowel disease (IBD) is transitioning from holistic community-level associations toward causal validation of individual bacteria and their metabolites. However, existing causal evidence is dispersed across diverse pathways and experimental models, lacking systematic integration and comparative assessment of evidence strength. Building on the concept of grading causal evidence in microbiome studies, we extend this approach into a five-tier progressive validation framework that covers the entire causal chain—from initial association discovery to the identification of specific effector metabolites. Using this framework, we systematically collate the single-bacterium–metabolite–host target axes that have been validated by high-level experimental methods. Focusing on four major pathways—AhR/tryptophan metabolism, bile acid/nuclear receptor signaling, short-chain fatty acid/vitamin/energy metabolism, and cell death-related pathogenic mechanisms—we integrate the molecular mechanisms through which specific bacteria and their causally linked metabolites regulate intestinal barrier integrity, immune responses, and inflammation resolution. We further discuss the modulatory influences of host genetics, diet, and medications on these axes, as well as therapeutic strategies such as live bacterial preparations, engineered probiotics, and postbiotics. Causal studies on individual bacteria and metabolites in IBD have progressed from isolated mechanistic discoveries towards systematic consolidation. It should be acknowledged, however, that most of the high-level evidence summarized in this review derives from animal models and preclinical studies, whereas robust human validation remains limited. In addition, protective mechanisms appear to considerably outnumber pathogenic ones, suggesting a marked imbalance in the current evidence base. To bridge the gap between mechanistic validation and clinical application, future research should focus on validating causal chains from animal models in well-designed human cohorts, exploring pathogenic mechanisms that drive disease onset, and clarifying interactions among different metabolites. By synthesizing available evidence, identifying key research gaps, and acknowledging translational limitations, this review aims to provide a foundational reference for researchers, while recognizing that further translational efforts are needed to move these preclinical findings toward clinical application.

## 1. Introduction

Inflammatory bowel disease (IBD), which mainly includes Crohn’s disease (CD) and ulcerative colitis (UC), represents a group of refractory digestive disorders characterized by chronic intestinal immune-mediated inflammation [1,2]. Currently, there appear to be two major challenges in the clinical management of IBD. First, disease subtyping and activity assessment rely heavily on endoscopy; however, as an invasive procedure, colonoscopy is not convenient for frequent monitoring [3]. In addition, currently available peripheral blood and fecal biomarkers lack sufficient specificity, and non-invasive stratification tools remain limited [4]. On the other hand, approximately 30–40% of patients with moderate-to-severe disease show primary or secondary loss of response to biologics, and individualized intervention strategies are not yet well established [5]. These issues may be approached from the perspective of the gut microbiota. Alterations in microbial composition and metabolic function could serve as potential sources of non-invasive biomarkers for disease stratification and activity assessment [6,7]. Moreover, differences in baseline microbiota among patients might affect drug metabolism and immune responses, thereby partly explaining inter-individual variability in biologic efficacy [8,9,10]. Current clinical dilemmas may not be fully explained solely by host immunity or intestinal mucosal pathology; therefore, there is a growing need to explore new avenues from the perspective of microbiota–host interactions.

Currently, gut microbiota dysbiosis has been implicated as one of the key drivers in the pathogenesis and progression of IBD [11]. Early studies based on 16S rRNA sequencing revealed dysbiotic features in the gut microbiota of IBD patients, primarily characterized by a reduction in short-chain fatty acid (SCFA)-producing bacteria and an expansion of opportunistic pathogens [12,13]. Subsequently, the widespread use of multi-omics technologies has progressively broadened the research perspective from compositional descriptions of microbial species toward dynamic functional analyses of metabolic activities, identifying alterations in pathways such as tryptophan metabolism and bile acid metabolism that are associated with IBD [14,15]. However, whether through metagenome-wide association studies or integrated multi-omics approaches, these analyses remain largely correlative, and it remains challenging to disentangle the temporal sequence and causal direction between microbial dysbiosis and intestinal inflammation. Despite these correlative limitations, the gut microbiome has been increasingly recognized as a promising therapeutic target in IBD, given its role as a metabolic organ that profoundly influences host physiology and disease progression [16]. Interventions such as fecal microbiota transplantation and broad-spectrum probiotics, which are based on whole-microbiota strategies, currently face challenges including inconsistent efficacy and substantial inter-individual variability in clinical trials [17], further pointing to the need to shift from community-level profiling toward validated single-bacterium–metabolite axes.

In this review, we tentatively define the single-bacterium–metabolite axis as the full molecular interaction sequence in which a particular gut bacterial strain, through synthesis, degradation, or transport of small-molecule metabolites, influences host intestinal receptors, immune signaling, and cell death pathways. This definition aims to shift the unit of analysis from the community level to specific strain–metabolite pairings, and to provide a common framework for integrating and grading causal evidence thereafter.

Over recent years, the progressive maturation of causal validation techniques, including gnotobiotic colonization, metabolite supplementation [18], and gene knockout [19], has made it possible to move specific bacterial strains and their effector metabolites from correlative associations toward causal identification. However, these causal findings remain scattered across different pathways and experimental models, and there appears to be a lack of unified criteria for evidence assessment and systematic comparative analysis within the field. Most existing reviews on the gut microbiota in IBD have tended to focus on a single signaling pathway or a particular class of intervention strategies; relatively few have addressed multiple pathways while also covering both protective and pathogenic mechanisms. Moreover, many reviews have not clearly differentiated the levels of evidence derived from different sources; they often include data from cohort correlation analyses, animal intervention experiments, and gene editing validation studies within the same narrative framework, which may make it difficult to objectively reflect the actual strength of the conclusions from each study. In addition, the link between basic mechanistic findings and clinical translation has long received limited attention. The practical bottlenecks that hinder the translation of bacterial strain and metabolite targets into noninvasive diagnostics or live bacterial therapeutics, such as from causal confirmation in animal models to efficacy validation in humans and from fully characterized strains to industrially manufacturable products, remain largely unaddressed and have not yet been systematically integrated or evaluated.

In response to the above limitations, this review first proposes a grading framework with five tiers, progressing from multi-omics association findings to causal validation. The framework is intended to offer a unified reference scale for assessing the strength of evidence in current IBD microbiome studies. Before applying this framework, however, it is worth clarifying how it relates to existing methodological tools in the field.

Chaudhari et al. proposed the causal funnel model in Nature Chemical Biology in 2021 [20], which provides a classic general framework for this field. Starting from association studies, the model progresses through germ-free and antibiotic-treated animal models and fecal microbiota transplantation, ultimately focusing on the identification of single strains and causal molecules. It systematically describes the typical experimental progression of causal research in the microbiome field.

Building upon this foundation, our five-tier framework is not a simple transplantation of that model but offers refinements in three key aspects. First, Chaudhari et al.’s model tends to be descriptive, summarizing what researchers typically do, whereas our framework is more prescriptive, specifying what criteria should be met to achieve a given level of evidence. Second, their evidence grading relies primarily on experimental type, whereas we explicitly require that at least one IBD-relevant pathological endpoint, namely barrier function, immune homeostasis, or cell death, be assessed for evidence grading. In this sense, our framework is specifically tailored to IBD, while their model serves as a general framework applicable to multiple diseases.

It should also be acknowledged that any framework has its scope and limitations. Currently, our framework is literature-based and has not yet been tested through prospective application. The assignment of evidence levels may involve some subjectivity, particularly when primary studies employ mixed experimental designs. Regarding applicability, although designed for IBD, the framework may be adaptable to other immune-mediated inflammatory diseases through appropriate modification of pathological endpoints. Regarding reproducibility, consistent application of the framework likely depends on transparent reporting of experimental methodologies in primary studies, which we suggest deserves attention in future research. We further acknowledge that reviewer variability may arise when applying the framework to heterogeneous study designs. To minimize this, we applied the predefined criteria uniformly. Future expert consensus or Delphi-based validation would be valuable to further strengthen the framework’s utility.

Based on this framework, we concentrate on four major pathways, namely AhR/tryptophan metabolism, bile acid/nuclear receptor signaling, short-chain fatty acid/vitamin/energy metabolism, and cell death and pathogenic mechanisms. We then systematically synthesize the single-bacterium, metabolite, and host target axes that have reached higher levels of causal validation, and we also perform a comparative analysis across these pathways regarding the completeness of validation and existing research gaps. Unlike previous reviews that concentrate on a single pathway, the present review uses the strength of causal evidence as its core thread throughout. This approach is intended to help researchers quickly locate high-confidence targets and key knowledge gaps, and to provide a practical roadmap for moving IBD microbiome research from correlative description toward clinical translation that is driven by mechanistic understanding.

### Literature Search Strategy

A literature search was performed in PubMed and Web of Science up to July 2026 using combinations of keywords related to IBD, the gut microbiota, microbial metabolites, and causal validation. The following keywords were used: “inflammatory bowel disease” or “IBD”, “Crohn‘s disease”, “ulcerative colitis”, “gut microbiota” or “gut microbiome”, “microbial metabolites”, “tryptophan”, “bile acid”, “short-chain fatty acid”, “causal”, “gnotobiotic”, “colonization”, “metabolite supplementation”, and “gene knockout”. Studies were included if they met the predefined criteria for Level 4 or Level 5 evidence as described in Section 3. Reference lists of retrieved articles were also screened for additional relevant studies. Given the narrative nature of this review, the selection of studies was guided by conceptual relevance rather than exhaustive systematic retrieval, and the included studies are intended to be representative rather than comprehensive.

## 2. Omics-Based Dimensions of Changes in the Microbiota

The focus of gut microbiota research appears to be shifting from describing taxonomic composition toward analyzing functional dynamics. The combined use of metagenomics, metatranscriptomics, metaproteomics, metabolomics, and viromics provides a systematic overview of interactions between the host and microbes in IBD. These findings constitute the evidence base at the correlational level, and their core value may lie in identifying potential candidate pairs of single bacteria and their associated metabolites.

### 2.1. Metagenomics: Moving from Taxonomic Composition Toward Functional Profiling

Metagenomics, based on shotgun sequencing of all microbial DNA from fecal or mucosal samples, can simultaneously reveal taxonomic composition, gene abundance, and genetic variations at the strain level [21]. Studies based on metagenomics have indicated that the gut microbiota of IBD patients not only exhibits dysbiosis at the species level, but also appears to be accompanied by widespread genomic structural variations [22].

One study analyzed fecal samples from 40 IBD patients and 47 healthy controls, and found that the IBD group had significantly lower alpha diversity, with enrichment of proinflammatory genera such as *Fusobacterium* and *Morganella*, and a marked reduction in commensals such as *Ruminococcus* and *Agathobacter*, which are known to produce short-chain fatty acids. At the functional level, the healthy control group was enriched in carbohydrate-active enzymes such as GH3 and GH44 that are involved in polysaccharide degradation, suggesting a greater capacity for dietary fiber utilization. In contrast, the IBD group was dominated by GT50 and GH6, which are related to bacterial cell wall synthesis, implying that under host inflammatory stress, the metabolic priority of the microbiota may have shifted from nutrient acquisition toward stress adaptation [23]. These differences at both taxonomic and functional levels may offer candidate clues for the subsequent screening of potentially pathogenic single bacteria and associated metabolic pathways.

In addition to conventional shifts in nutrient metabolism, metagenomic gene counts may also reveal associations between microbial gene abundances and clinical heterogeneity as well as drug efficacy [24,25]. One study found that IBD patients carrying multiple microbial acetyltransferase genes had an increased risk of treatment failure with mesalamine [26], suggesting that metagenomics may not only be used for screening associations at the species and functional levels, but may also identify microbial genes directly related to drug metabolism, thus providing candidate targets at the functional gene level for subsequent causal validation.

### 2.2. Metatranscriptomics and Metaproteomics: Insights into Functional Activity of the Gut Microbiota

Metatranscriptomics directly measures the abundance of microbial gene transcripts, thereby overcoming the limitation of metagenomics, which only reveals potential functional profiles [27]. A longitudinal cohort study of IBD found that even when species abundances remained relatively constant, the transcriptional activity of metabolic pathways in key commensals varied with host disease activity [28]. Such changes at the transcriptional level could precisely reveal when and where effector molecules of the single-bacterium–metabolite axes are activated in the host, providing temporal evidence for observing how microbial functions change with disease progression. Further integrated analysis of longitudinal metatranscriptomics and metagenomics suggested that this integrated omics approach could produce functional dynamic maps of the microbiome with high resolution, suggesting that the research focus may have shifted from static compositional description toward dynamic functional analysis [29].

While metatranscriptomics may reveal functional tendencies, metaproteomics, through direct detection of protein expression and post-translational modifications in both microbes and the host, could pinpoint the direct effectors of phenotypes. In one integrative multi-omics study, researchers systematically identified more than 340,000 microbial protein families with potential biological activity in the gut of IBD patients, about half of which had lacked functional annotation previously [30]. That study further demonstrated, through subsequent experiments, that the immunogenicity of *Enterobacteriaceae* strains exhibited notable strain specificity and that the secreted von Willebrand factor homologs could promote biofilm formation in a manner that depends on mucus, thereby aggravating intestinal barrier damage. These differences observed at the protein level may constitute the molecular basis for the core biological effects of the single-bacterium–metabolite–host interaction axis, and the underlying causal mechanisms could be further validated through gene knockout or protein neutralization experiments.

### 2.3. Metabolomics: The Ultimate Representation of Functional Output

Metabolomics, as a direct output of microbial functional activity, may serve as a molecular bridge connecting gut dysbiosis to host pathophysiology [31]. Changes in metabolite levels can reflect microbial metabolic output, and also represent a critical step in moving from correlation toward causal validation. However, the independent clinical predictive value of metabolomics still needs to be assessed with caution. A prospective study that integrated metagenomics and untargeted metabolomics in an IBD cohort receiving biologic therapy found that althoughseven core metabolites and the ratio of primary to secondary bile acids differed significantly between responders and non-responders, the area under the curve (AUC) of the metabolomics model alone was only 0.70, comparable to that of the clinical model (0.71), and reproducibility across cohorts was low [32]. This negative finding may suggest that relying solely on static metabolite abundance associations may be insufficient for building generalizable predictive models, and that causal chains may need to be verified through gnotobiotic colonization or precise metabolite supplementation experiments.

Despite encountering limitations in predicting treatment response at later disease stages, metabolomics might still hold unique value for early disease warning and elucidation of microscopic mechanisms [33]. On one hand, metabolomics can capture subtle metabolic changes that occur before disease onset in the host. In a prospective nested case–control study involving 5122 healthy first-degree relatives of patients with CD, elevated serum quinolinic acid and reduced ascorbic acid and isocitric acid were found to independently predict the risk of developing CD within the subsequent 5 years. Notably, quinolinic acid showed a positive correlation with the abundance of *Ruminococcus torques*, and mediation analysis preliminarily suggested a potential forward causal pathway from single bacteria to metabolites and then to disease risk [34].

On the other hand, precision metabolomics may help pinpoint functional pathways and intervention targets within specific bacterial axes. Through integration of metagenomics and metatranscriptomics, researchers observed that expression of LuxR-type receptors in the genus *Bacteroides* was markedly altered in IBD patients, suggesting that microbial quorum-sensing small molecules may participate in signaling communication that mediates microbe–host interactions [35]. Further tracing using stable isotope-resolved metabolomics (SIRM) provided evidence that gut microbes can actively synthesize polyamines through the arginine-to-agmatine-to-spermidine pathway, and that Bacteroides may serve as a key contributor to the elevated fecal polyamine levels observed in IBD patients [36]. These well-characterized metabolic pathway identifications may not only deepen our functional understanding of the single-bacterium–metabolite axes, but may also offer precise molecular targets for future interventions that use synthetic biology approaches to selectively knock out or complement specific metabolic genes, potentially blocking the progression of intestinal inflammation.

### 2.4. Gut Virome: A Potential Role in IBD

In addition to bacteria, the role of gut viruses, including phages and eukaryotic viruses, in the pathological progression of IBD has received growing attention [37]. A multi-omics study that combined metagenomics, metaviromics, and metatranscriptomics systematically compared, for the first time, phage communities at the interface between colonic mucosa and lumen with those in feces. The results showed that mucosal phage communities differed markedly from fecal viromes and were enriched in crAss-like phages that were difficult to detect in feces. Further integrated analysis revealed that only a fraction of viruses were transcriptionally active, and that the presence of integrase genes did not reliably predict the lysogenic state, whereas metatranscriptomic data could more accurately determine the actual lifestyle of phages [38]. This suggests that phages may indirectly modulate the output of the single-bacterium-and-metabolite axis by lysing specific bacteria.

In addition, the role of eukaryotic viruses in early IBD pathogenesis is also emerging. Researchers performed metatranscriptomic analysis on intestinal mucosa from young, newly diagnosed, untreated IBD patients, and found that transcripts of the Hepadnaviridae family were significantly elevated in UC patients, while Hepeviridae were elevated in CD patients, and several virus families derived from plants or insects were markedly reduced, suggesting that the heterogeneous expression profiles of specific eukaryotic gut viruses may be involved in early IBD pathogenesis [39].

In summary, multi-omics technologies have provided abundant correlational evidence linking microbiota, genes, metabolites, and viruses to IBD, and have depicted a candidate molecular network for the single-bacterium–metabolite axes. However, correlation does not necessarily imply causation. Identifying potential causal associations frommulti-omicss findings would require further validation through targeted experiments. These efforts may offer more definitive intervention targets for precision therapy of IBD.

## 3. Experimental Methodology for Establishing Causality

**Figure 1:**
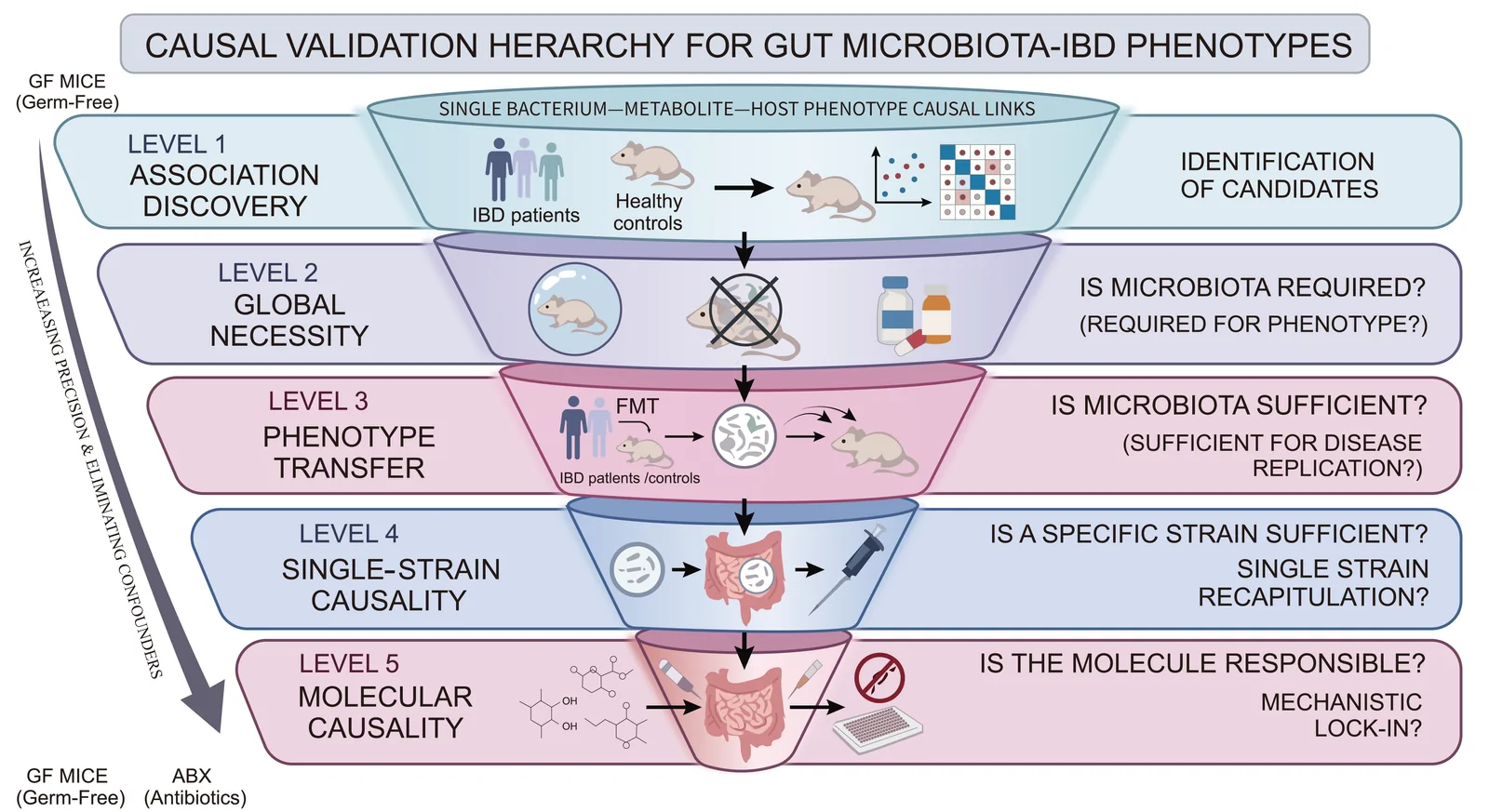
The five-level causal validation framework for the single-bacterium-and-metabolite axis. The framework divides evidence into five progressive levels (Level 1 to Level 5) according to the strength with which experiments establish the causal chain. Arranged from bottom to top, higher levels correspond to greater precision in identifying the causal link.

To systematically organize existing causal evidence on single-bacterium-and-metabolite axes in IBD, we propose a five-level grading framework based on the causal funnel concept [20] but adapted to the IBD setting with disease-specific pathological criteria, as detailed in the Introduction. The framework assigns evidence levels according to the extent to which experimental designs can confirm the causal link between a strain and its metabolite. It is divided into five levels, from Level 1 to Level 5 (Figure 1), with Levels 4 and 5 being the focus of systematic integration in this review. Detailed definitions and criteria for each level are provided in Section 3. It should be noted that this grading framework is intended to serve as a reference standard for the literature included in our review; it is not meant to replace causal inference at the population level from Mendelian randomization studies or prospective clinical intervention cohorts, nor should it be viewed as a universal gold standard in the field.

Level 1 is association discovery, which aims to identify differential microbiota and metabolites that are consistently associated with disease by comparing IBD patients and healthy controls. This level mainly relies on 16S rRNA sequencing, metagenomics, and untargeted or targeted metabolomics, combined with prospective cohort designs and network analysis of microbe metabolite associations [40], to identify strongly correlated strain–metabolite pairs as candidate targets for subsequent validation. However, association results from a single cohort may fluctuate due to factors such as sample size, disease subtypes, and medication history, and integrative analysis across cohorts has become a necessary step to enhance the robustness of associations [41]. Association studies can answer what changes in the microbiota and metabolites occur in disease, but they can neither distinguish the direction of causality nor exclude confounding effects; therefore, they represent the starting point rather than the endpoint of causal validation.

Level 2 is validation of global necessity, which aims to test whether the microbiota as a whole is necessary for the IBD phenotype. This level uses germ-free (GF) animals and broad-spectrum antibiotic depletion models to observe whether IBD-related phenotypes and metabolite profiles change in the absence of the gut microbiota. Consistent findings suggest that complete removal of the microbiota can alleviate DSS-induced acute inflammation, but may also severely impair intestinal epithelial barrier integrity, leading to an unexpected increase in mortality [42]. Meanwhile, antibiotic elimination of the microbiota abolished the protective effect of parthenolide (PTL) against colonic inflammation, further supporting, from an intervention perspective, the necessity of the overall microbiota in this protective effect [43]. These findings indicate that the microbiota as a whole plays a critical role in IBD. However, this type of experiment only demonstrates the role of the community, and cannot pinpoint which specific bacterium or metabolite is responsible. In addition, antibiotic treatment itself may affect host immune cells, and GF animals have inherent developmental defects. Therefore, although Level 2 confirms the involvement of the microbiota as a whole, it is insufficient to identify the specific members of the causal chain.

Level 3 is phenotype transfer validation, which aims to investigate whether dysbiosis can directly drive IBD phenotypes independently of host genetic background. This level uses fecal microbiota transplantation (FMT) between different species or within the same species to transfer the entire microbiota from IBD patients or healthy controls into GF recipient animals, and then tracks metabolic changes and inflammatory phenotypes after transplantation. Studies have shown that transplanting the IBD patient microbiota into GF Smad3^−^/^−^ and wild-type mice can induce intestinal inflammation in recipients, and the severity is modulated by host genetic background, suggesting that dysbiosis is a pathogenic driver independent of host genes [44]. However, the efficacy of FMT in complex diseases is highly heterogeneous. The colonization efficiency of donor microbiota and expression of metabolic functions in recipients vary greatly, and the same donor microbiota can produce markedly different phenotypes in recipients with different genetic backgrounds [45]. Although Level 3 is stronger than Level 2, it still represents evidence at the community level, and cannot distinguish whether the effect is due to a specific strain, a single metabolite, or the synergistic action of multiple bacteria. However, the first three levels always use the microbiota as a whole as the operational target, and cannot attribute the effect to specific strains or molecules. To establish actionable molecular targets, causal validation needs to further focus the unit of analysis on individual strains and single molecules.

Level 4 is single-bacterium causal validation, which aims to test whether a specific strain, in the absence of other microbiota, is sufficient to reproduce particular metabolic changes and IBD-related phenotypes. Key techniques for this level include isolation and pure culture of the target strain, colonization of GF or antibiotic pretreated mice with a single strain, and simultaneous monitoring of metabolomic and phenotypic changes after colonization. Taking *Faecalibacterium prausnitzii* as an example, early studies only found an association between its abundance and the risk of postoperative recurrence in CD; subsequent colonization experiments with a single strain in GF mice confirmed that its colonization could upregulate Foxp3^+^ Treg cells and inhibit Th17 differentiation through butyrate production, thereby establishing the protective effect of this bacterium [46]. Similarly, single-strain colonization experiments with *Ruminococcus gnavus* revealed strain-specific pathogenicity, with nonencapsulated isolates causing more severe intestinal inflammation, and significant differences in tryptophan metabolic profiles across different strains [47]. The core contribution of Level 4 is that it narrows the causal chain down to a single strain. However, after colonization, the strain may simultaneously produce multiple metabolites, and single-strain colonization itself cannot distinguish which one or which combination of these metabolites mediates the phenotypic changes. In other words, although Level 4 confirms a causal relationship from a strain to a phenotype, it has not yet decomposed this causal chain further to the molecular level.

Level 5 is molecular causal validation, which aims to test whether a single molecule, in the absence of any live bacteria, is sufficient to reproduce or block IBD phenotypes through direct administration of candidate metabolites. This level uses various approaches, including direct metabolite supplementation, bacterial strains with knockout of metabolite synthesis genes, chemical inhibitors, and in vitro cell models, to establish the identity of effector molecules. Among these, the metabolite replenishment experiment is the most direct method of validation at this level. Taking indoleacrylic acid (IA) as an example, prophylactic oral administration of IA was found to significantly reduce the severity of DSS-induced colitis and increase mucus layer thickness. Meanwhile, the abundance of the IA synthesis gene cluster was markedly reduced in the metagenomes of IBD patients. The combination of metabolite supplementation and functional gene deficiency in the microbiota provided bidirectional evidence that fully established the causal chain [48]. Similarly, the protective effect of *Lactobacillus reuteri*, which produces indole 3 acetic acid (IAA), was lost after knockout of the *iaaM* gene, while exogenous IAA supplementation could restore the effect. This reverse validation paradigm, combining gene knockout with metabolite supplementation, is considered the gold standard for Level 5 evidence [49]. Metabolite supplementation experiments also have methodological limitations: the administered doses may far exceed physiological concentrations, and oral bioavailability is affected by intestinal absorption and hepatic first-pass metabolism; therefore, metabolite exposure levels in animal models may not be directly comparable to those produced in situ by the microbiota. Thus, Level 5 evidence may need to be complemented with multiple model systems and dose–response analyses to exclude nonphysiological off-target effects.

In our integration criteria, studies at Level 4 are eligible for inclusion if they meet the following conditions: use of germ-free or antibiotic-depleted animals to exclude interference from the background microbiota, quantitative measurement of metabolic changes after colonization, and simultaneous assessment of at least one IBD-related phenotype, such as histological score, inflammatory cytokines, or barrier function indicators. For Level 5, the criteria require that metabolite supplementation experiments reproduce a protective or pathogenic phenotype in at least one colitis model, or that a strain with knockout of the metabolite synthesis gene is constructed and the effect is shown to be restored by supplementation, or that host targets of the metabolite are confirmed using receptor knockout mice or chemical inhibitors. These criteria were applied consistently during literature screening. When a single study provided evidence spanning multiple levels, for example, both strain colonization (Level 4) and metabolite supplementation (Level 5), it was classified according to the highest level of validation achieved, provided that the experimental design met the corresponding criteria for that level. This approach ensured that the evidence level assigned to each study reflected its strongest causal contribution.

Based on these criteria, only studies reaching Level 4 or Level 5 are included in Section 4.

## 4. Molecular Mechanisms of the Single-Bacterium–Metabolite Axes in Regulating Host Intestinal Fate

### 4.1. The AhR–Tryptophan Metabolite Axis

**Figure 2:**
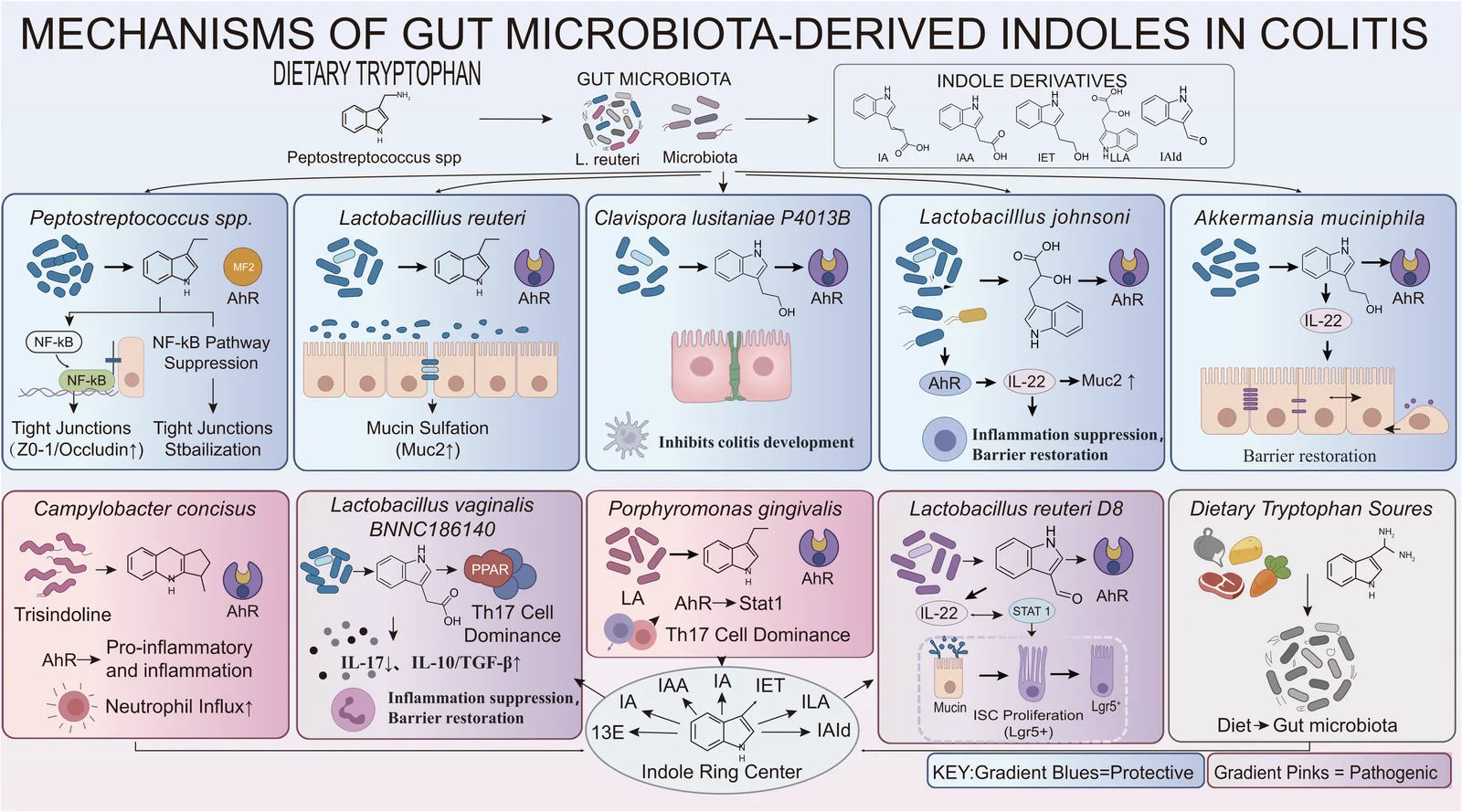
Molecular mechanisms of gut microbiota-derived indole metabolites in regulating colitis. Dietary tryptophan is metabolized by the gut microbiota into various indole derivatives. These metabolites can exert protective or pathogenic effects through activation of AhR or related pathways (blue and purple: protective; pink: pathogenic). Arrows indicate the correspondence between metabolites and their receptors, as well as the direction of signal transduction. In this figure, arrows “→” denote activation or positive regulation, while “↑” and “↓” indicate upregulation and downregulation of downstream molecules, respectively.

Tryptophan-derived indole compounds produced by the gut microbiota are major endogenous ligands for the aryl hydrocarbon receptor (AhR) [50]. The role of this pathway in maintaining intestinal epithelial barrier and mucosal immune homeostasis has been widely recognized, and it appears to be one of the areas where causal evidence for the single-bacterium–metabolite axis is relatively concentrated in IBD (Figure 2).

Several causal chains in this pathway have reached Level 4 or Level 5 validation (Table 1), with strategies including metabolite supplementation, gene knockout, and receptor blockade. Notably, trisindoline from *Campylobacter concisus* is among the few pathogenic molecules validated at Level 5, driving proinflammatory responses through AhR activation [51]. The opposing effects of protective versus pathogenic AhR ligands suggest that functional outcome depends on ligand structure rather than receptor binding alone. Overall, the tryptophan–AhR axis shows relatively rich causal evidence and strain–metabolite diversity.

From the perspective of receptor dependency, the metabolites in the above Level 5 chains can be divided into two categories. The protective effects ofIAA, indole-3-lactic acid (ILA) and indole-3-ethanol (IET) appear to depend on AhR, as their effects can be blocked by AhR antagonists or gene knockout [49,52,53]. In contrast, the protective effect of IA persists even after AhR blockade, and current evidence points to activation of the Nrf2 pathway as the mechanism underlying its barrier-protective function [47], representing an AhR-independent protective mechanism. In addition, 3-indoleacrylic acid derived from *Lactobacillus vaginalis* has been reported to exert its effects through PPAR signaling [61], suggesting that the same metabolite may act through different receptors depending on the bacterial strain context. In contrast to these protective mechanisms, trisindoline from *C. concisus*, although it also activates AhR, appears to drive proinflammatory rather than protective responses [50]. These observations suggest that determining whether a tryptophan metabolite activates AhR alone is insufficient to predict the direction of its functional effects.

The observation that different AhR ligands can produce opposing effects may be related to ligand-induced conformational changes in the receptor and subsequent differences in downstream transcriptional output. Although IAA, ILA, IET, and trisindoline can all bind to AhR, the gene expression programs they trigger appear to differ [48,51,52]. Activation of the receptor may be only the first step; which downstream genes are mobilized after activation may be the key determinant of functional outcome. The interaction patterns of different indole metabolites with key residues in the AhR ligand-binding domain may differ, and such differences could influence the magnitude and direction of receptor conformational changes, thereby affecting the recruitment preference of coactivators or corepressors. From a biological perspective, this ligand-dependent functional divergence may reflect a mode of regulation between the gut microbiota and the host immune system: the microbiota, by producing ligands with different structures, may deliver distinct immune signals while activating the same receptor. This also suggests that when evaluating AhR as a therapeutic target, focusing on the specific transcriptional output driven by ligand–receptor binding may be more meaningful than simply classifying molecules as agonists or antagonists.

The AhR-independent mechanism of IA also warrants attention. If its protective effect is indeed mediated through Nrf2, then IA or structurally related molecules could serve as an alternative class of candidate compounds alongside AhR agonists, and might be applicable to IBD subtypes with impaired AhR signaling or suboptimal AhR activation. Taken together, the protective mechanisms of tryptophan metabolites do not appear to constitute a single pathway, but rather a regulatory network composed of multiple metabolites and multiple signaling branches. Understanding the operational logic of this network may help to provide a foundation for the design of subsequent precision intervention strategies.

### 4.2. Bile Acid-and-Nuclear Receptor Signaling Axis

The gut microbiota converts primary bile acids synthesized by the liver into secondary bile acids through reactions including deconjugation, dehydroxylation, and epimerization. These secondary bile acids act as ligands for the farnesoid X receptor (FXR), TGR5, and the NOD-like receptor family pyrin domain-containing 3 (NLRP3) inflammasome, and play key signaling roles in lipid metabolism, inflammatory responses, and intestinal barrier regulation [62]. Secondary bile acid reduction and FXR signaling suppression are commonly observed in IBD patients [63]. Recent causal validation studies have successfully linked the effects in this pathway to specific strains and their metabolites.

FXR is highly expressed in the ileum but at lower levels in the colon. Ileal FXR activation mainly induces antimicrobial peptides to limit bacterial overgrowth, whereas colonic FXR appears to be more involved in immune modulation [64]. This segment-specific difference suggests that defective FXR signaling in the ileum and colon may lead to distinct pathological outcomes, with bacterial translocation being more prominent in the former and local inflammation amplification in the latter.

Key Level 4 and Level 5 findings in this pathway are summarized in Table 1. Among them, *Akkermansia muciniphila* has been shown to activate FXR through restoration of DCA and UDCA levels, thereby inhibiting unspliced *XBP1* to alleviate endoplasmic reticulum stress [58]; *Candida metapsilosis M2006B* secretes acyclic sesquiterpenoids that act as FXR agonists [59]; and *Odoribacter splanchnicus* suppresses NLRP3-GSDMD-mediated NET formation through elevation of LCA levels [60]. In addition, LCA and DCA can inhibit NLRP3 inflammasome activation through TGR5 [65], suppress Th1 differentiation through VDR [66], and inhibit the NF-κB pathway through PXR [61]. However, the cooperative or compensatory relationships among these multiple receptors remain unclear and represent an important direction for future research.

Compared with other axes, a notable feature of the bile acid pathway is the dose-dependent and context-dependent direction of its effects. For example, DCA exerts protective effects by inhibiting excessive FXR activation in the context of *Lactiplantibacillus plantarum AR113* intervention [67], whereas in high-fat-diet models, elevated DCA may exacerbate colonic inflammation by inducing ferroptosis through the HIF-2α/DMT1 pathway [68]. This observation suggests that translational application of the bile acid pathway may require more precise dosage control and disease stage matching than the AhR axis.

In addition, this pathway shows clear crosstalk with other axes. DCA may influence AhR signaling intensity, while ferroptosis protection mechanisms are intertwined with GPX4 regulation in the energy metabolism axis [69]. These interactions enrich the network of microbe–host interactions, but also add complexity to clinical translation. At present, FXR agonists have entered clinical trials for metabolic diseases [70], but further evidence is needed for their application in IBD.

### 4.3. Short-Chain Fatty Acid/Vitamin/Energy Metabolism

This section includes 11 Level 4 and Level 5 causal validation studies (Table 2). Multiple chains have reached Level 5, including spermidine (SPD) production by *Akkermansia muciniphila* [70], R-equol production by *Bifidobacterium pseudolongum* [71], N-acetylspermidine production by *A. muciniphila* [72], methylnicotinamide (MNA) production by *Lacticaseibacillus rhamnosus GG* [73], butyrate production by Bacillus velezensis [74], and N-carbamylglutamate (NCG) production by *Limosilactobacillus reuteri* [75].

These Level 5 causal chains can be functionally categorized into three types: short-chain fatty acid energy supply, vitamin-derived signaling regulation, and amino acid-derived metabolic regulation. They represent core modules through which the microbiota regulates host basal physiological homeostasis, and also illustrate the feature that a single strain can mediate multiple metabolic axes simultaneously.

Compared with the tryptophan and bile acid pathways, the mechanisms involved in this section are relatively more complex. One important reason is that most metabolites in this section lack a single signature receptor. As discussed earlier, tryptophan metabolites can directly bind to AhR, and bile acid metabolites are primarily centered on FXR; both pathways have well-defined core receptors, allowing causal validation to be directly established through receptor blockade or gene knockout experiments. In contrast, among the metabolites covered in this section, only butyrate has relatively well-characterized receptors (FFAR2/FFAR3) [74], while the direct receptors for other metabolites, such as MNA [73], SPD [77], and NCG [75], have not been fully identified. The six Level 5 chains in this section are relatively more specific, exemplified by *L. rhamnosus GG* and MNA [73], and *B. velezensis* and butyrate [74], with clear targets that allow causal relationships to be locked through receptor blockade or gene knockout. For the remaining cases that do not reach Level 5, there appear to be multiple targets, with the same metabolite potentially activating several receptors simultaneously; for instance, butyrate can both activate *GPR41/43* and inhibit *HDAC* [76], making it difficult to distinguish independent effects from synergistic ones.

This regulatory pattern, characterized by multiple molecules and the absence of a core receptor, may also limit the feasibility of clinical translation. Single-receptor-targeted drugs could exert effects through activation of AhR or FXR, but this section lacks a comparable core receptor. Current evidence is insufficient to determine whether supplementation with a single metabolite, such as butyrate or MNA alone, can fully recapitulate the regulatory effects achieved by strains like *A. muciniphila* that secrete multiple metabolites in situ and engage multiple pathways simultaneously. Furthermore, the concentration of exogenously supplemented metabolites may differ from the dynamic concentration gradients achieved during strain colonization. From a methodological perspective, future studies could consider a multi-receptor combination validation approach, using multi-gene knockout models to systematically dissect the core effector molecular combinations, thereby guiding the development of composite metabolite-based therapeutics.

### 4.4. Cell Death and Pathogenic Mechanisms in IBD

Unlike the preceding sections that focus on protective mechanisms, this section addresses two directions of causal chains with opposite effects (Table 2). Pathogenic strains can directly activate inflammatory pathways or induce host cell death through specific molecules, whereas protective strains alleviate inflammation by inhibiting ferroptosis, neutrophil extracellular trap formation (NETosis), or apoptotic pathways [82,83,86]. Ferroptosis, an iron-dependent form of programmed cell death driven by lipid peroxidation, has been implicated in intestinal epithelial damage in IBD. NETosis, on the other hand, disrupts tight junctions and activates inflammatory cascades through release of neutrophil extracellular traps [87].

In terms of pathogenic mechanisms, several causal chains have reached the level of single-bacterium validation, but their extension to the molecular level remains limited. For example, *Ruminococcus gnavus* activates TLR4 through its surface polysaccharides, promoting TNF-α and IL-8 secretion from dendritic cells [47]; however, the specific pathogenic polysaccharide has not yet been clearly identified. *Enterococcus faecalis* has been reported to hijack host *FABP2* to activate quorum sensing systems and promote its own overgrowth [83]; this mechanism involves host–protein and bacterial–signal interactions, but no microbiota-derived metabolite has been specifically implicated. At present, the only pathogenic chain that has reached Level 5 is that of *Parasutterella excrementihominis*, which produces succinate and 6-hydroxyhexanoic acid, activating SUCNR1 and GPR84 to induce NLRP3/GSDMD-mediated NETosis [84]. In contrast, the pathogenic effects of *R. gnavus* and *E. faecalis* still remain at the strain level, with their specific effector molecules yet to be identified.

Taken together, the evidence base for protective mechanisms is substantially greater than that for pathogenic mechanisms. This disparity may be partly related to the predominant experimental models currently used. Most IBD studies widely employ acute colitis models induced by DSS or TNBS [88]. These models first create chemical injury and then test whether interventions can promote resolution of inflammation. Thus, they are better suited for studying mechanisms related to how inflammation is resolved, but may be less capable of modeling how inflammation spontaneously initiates in human IBD. Long-term reliance on such models may inadvertently bias research toward protective mechanisms, as these experimental workflows are relatively straightforward and endpoints are well defined. In contrast, studies of pathogenic mechanisms require observation of spontaneous disease onset without relying on chemical injury, which involves more complex experimental designs and greater technical challenges.

From a clinical perspective, understanding the pathogenic mechanisms of IBD, including how the disease initiates and why inflammation persists, is equally important. Future studies that incorporate spontaneous inflammation models more closely resembling human pathology, or that appropriately shift the emphasis of causal validation toward pathogenic mechanisms, may help narrow this evidence gap.

## 5. Modulatory Factors of Microbiota–Metabolite–Host Interactions

### 5.1. Host Genetic Factors

Host genetic factors may modulate the single-bacterium–metabolite axis at three levels: microbial composition, metabolite production and conversion, and host target responsiveness to metabolites. Current evidence has accumulated varying degrees of findings at each of these levels.

At the level of microbial composition, IBD risk gene variants may shape the species structure of the gut microbiota. Individuals carrying the *ATG16L1 T300A* risk variant have been shown to exhibit selective expansion of specific taxa such as *Bacteroides ovatus* before the onset of tissue inflammation, accompanied by enhanced local Th1/Th17 responses in the colon [89]. In mice with intestinal epithelial-specific deletion of *Atg16l1*, a reduction in butyrate producers and an increase in proinflammatory bacteria were observed [90]. These findings suggest that the effect of genetic variants on microbial structure may already be established before inflammation is initiated.

At the metabolite level, host genetic deficiencies may alter the metabolic output of the microbiota. The aforementioned *Atg16l1*-deficient mice showed not only altered microbial composition, but also elevated fecal N-acetylglucosamine and reduced serum inositol and glucose, with these metabolic changes occurring in parallel with elevated colonic TNF-α and CXCL1 [90]. Similarly, *NOD2* loss-of-function variants, due to failure to bind GIV protein, may impair macrophage bactericidal activity, thereby exacerbating the vicious cycle of dysbiosis and inflammation [91]. However, studies that directly link specific genetic variants to particular effector metabolites remain relatively scarce, with most evidence currently confined to microbial composition or inflammatory phenotypes.

At the level of host target responsiveness, genetic variants may alter the sensitivity of the host to the same metabolite. Genome-wide association studies have identified more than 200 IBD susceptibility loci, which are enriched in key pathways including bacterial recognition (*NOD2*), autophagy (*ATG16L1, IRGM*), endoplasmic reticulum stress (*XBP1, AGR2*), and Th17 immune responses (*IL23R*) [92]. Variants in these genes may change the efficiency with which intestinal epithelial cells sense AhR ligands, FXR agonists [63], or butyrate [93], potentially leading to different intensities of inflammatory responses even under similar metabolite levels. Likewise, polymorphisms in FXR and AHR genes may affect the responsiveness threshold of intestinal epithelial cells to bile acids or indole metabolites [94]. However, direct evidence linking specific metabolites to host target genotypes in IBD is currently lacking, and most available data are limited to indirect inferences. This gap suggests that even when a given metabolite shows clear causal effects in animal models, the magnitude of its effects may still vary considerably among patients with different genetic backgrounds. This could represent the next layer of complexity to be considered when moving the single-bacterium–metabolite axis from causal validation toward clinical translation.

### 5.2. Environmental and Dietary Factors

Environmental factors, including diet and antibiotic exposure, may exert influences at different levels of the single-bacterium–metabolite axis by reshaping microbial composition or directly modulating metabolite levels.

At the level of microbial composition, a high-fat diet has been shown to significantly enrich opportunistic pathogens such as Clostridium in the gut, promoting excessive production of DCA. DCA may induce macrophage polarization toward a proinflammatory M1 phenotype through the M2-mAchR/TLR2 signaling axis, while also activating the intestinal epithelial HIF-2α/DMT1 pathway to trigger ferroptosis, synergistically exacerbating colonic inflammation [68]. Of note, DCA was also identified as a metabolite that was reduced following *Lactiplantibacillus plantarum AR113* intervention in the bile acid pathway [67]. The same molecule may exhibit opposite directionality under probiotic intervention versus high-fat diet conditions, suggesting that environmental modulation of metabolite levels could alter its pathological or protective properties. Clinical data have also shown that active UC patients consuming a high-fat diet present more pronounced ferroptotic features in colonic tissue, and that fat intake appears to be positively correlated with disease activity [95]. Even without altering total fat content, the processing method of oils may independently exert effects; interesterified palm oil, under normal lipid levels, has been reported to increase intestinal permeability, promote bacterial translocation, and disrupt tight junction protein expression, with barrier damage comparable to that of a high-fat diet itself, possibly related to microbiota-mediated changes in lipid metabolites [96]. In addition, the impact of antibiotic exposure may extend to subsequent generations through vertical transmission of the microbiota. Colonization of germ-free pregnant mice with a dysbiotic microbiota derived from penicillin-exposed donors led to sustained low diversity and unstable microbial states in offspring, accompanied by reduced production of secondary bile acids and short-chain fatty acids, and markedly increased colitis severity, in an IL-10-deficient context [97], suggesting that antibiotic-induced perturbations of microbial composition may be amplified across generations.

At the level of metabolite regulation, dietary intervention may directly alter the bioavailability of effector metabolites [98]. A low-fat, high-fiber diet in UC patients during remission not only reduced serum amyloid A levels, but also improved dysbiosis, with a decrease in Actinobacteria and an increase in Bacteroidetes, along with elevated relative abundance of the anti-inflammatory commensals *Faecalibacterium prausnitzii* and *Prevotella*, while fecal levels of proinflammatory saturated fatty acids were reduced [99]. The beneficial effects of this dietary intervention appear to contrast with the detrimental effects of a high-fat diet; the former increases the proportion of butyrate-producing bacteria and lowers proinflammatory lipid levels, whereas the latter enriches DCA-producing Clostridium and induces ferroptosis, indicating that the direction in which environmental factors shape the metabolite profile may determine whether the net effect is proinflammatory or anti-inflammatory.

### 5.3. Drug–Microbiota Bidirectional Interplay

The preceding section discussed how external factors such as diet and antibiotics may influence the starting point and transmission of the causal axis by altering microbial composition and metabolite profiles. However, in the real-world treatment of IBD patients, the relationship between drugs and the gut microbiota is not unidirectional. Drugs may act on the microbiota, while the microbiota can conversely modify drug activity through its rich repertoire of metabolic enzymes. This bidirectional interaction may also reshape the metabolic output of the microbiota itself, thereby affecting the strength of the effects along the aforementioned causal axes [100,101]. Understanding this two-way relationship may help to explain why the same causal axis may produce different therapeutic responses in different patients.

The gut microbiota encodes a large set of enzymes capable of chemically modifying various orally administered drugs [102]. Microbial inactivation of digoxin and levodopa has been reported previously [103,104]. In recent years, this phenomenon has also been validated in the context of IBD therapeutics. 5-aminosalicylic acid (5-ASA) is a commonly used first-line drug for IBD, but its clinical response rate is less than 50% [105]. By integrating metagenomic, metatranscriptomic, and metabolomic data, Mehta et al. identified 12 gut microbial acetyltransferases that can convert 5-ASA into the inactive form N-acetyl-5-ASA. Among these, a thiolase from the uncultured Firmicutes CAG:176 and an acyltransferase from *Faecalibacterium prausnitzii* were both shown to possess this activity. Patients carrying three or more such enzyme genes were found to have a significantly higher risk of requiring glucocorticoid therapy after 5-ASA use, with adjusted odds ratios ranging from 2.58 to 3.24, and this association was validated in an independent cohort [26]. Of note, the abundance of these enzyme genes did not differ significantly between 5-ASA users and non-users, suggesting that they represent intrinsic microbial features rather than drug-induced changes. Thus, they may serve as potential biomarkers for pre-treatment stratification.

The microbiota can also activate prodrugs. Sulfasalazine is cleaved by intestinal azoreductases to release 5-ASA, representing a classic example of microbiota-dependent prodrug activation [106]. Conversely, microbial metabolism may sometimes enhance drug toxicity. For instance, the active metabolite SN-38 of irinotecan can be reconverted into a toxic form by gut bacterial β-glucuronidases, leading to severe diarrhea [107]. This mechanism may be particularly relevant in IBD patients, as increased intestinal permeability and altered microbial structure may further amplify this toxic pathway. On one hand, microbial drug metabolism may directly alter drug efficacy; for example, azoreductase activation of sulfasalazine and β-glucuronidase reactivation of SN-38 suggest that the distribution of metabolic enzyme genes in the baseline microbiota could be an important source of variability in treatment response. On the other hand, metabolites consumed or generated during microbial drug metabolism may indirectly perturb the regulation of endogenous metabolites on host targets, thereby influencing the strength of effects along the causal axis.

However, most current studies still focus on enzyme–drug or drug–efficacy relationships, and relatively few have systematically evaluated the specific impacts of drug metabolism on microbial composition, metabolite profiles, or host target responsiveness. This gap suggests that when moving from causal validation toward clinical translation, the presence of therapeutic drugs should be taken into account as part of the dynamic regulatory network of the single-bacterium–metabolite axis.

## 6. Therapeutic Strategies Based on the Single-Bacterium–Metabolite Axis

### 6.1. From Causal Targets Toward Live Biotherapeutic Products

The preceding sections have reviewed causally validated strains and their effector metabolites across four pathways. These findings may provide clear molecular targets for the development of live biotherapeutic products. However, the translational paths for different strains are not identical. Below, we use four representative candidate strains as examples to illustrate their respective translational bases and directions for future research.

*Akkermansia muciniphila* appears to be the most advanced among the four candidates in terms of translational progress. This strain has been supported by three independent causal pathways for its protective effects: tryptophan–AhR (I3E) [55], bile acid-FXR (p-HPAA/DCA and UDCA) [58], and energy metabolism (SPD and N-acetylspermidine) [36]. At the clinical level, pasteurized *A. muciniphila* has completed a Phase II trial in overweight/obese individuals (NCT05114018), and a Phase II trial of the live product AKK-WST01 in patients with type 2 diabetes has also been completed (NCT04797442). For IBD, a Phase IV trial in combination with infliximab was initiated in 2026 (NCT07415473). A key task at present may be to clarify which of the three pathways plays a dominant role in humans, as this may determine whether the subsequent development should proceed as a live product or a postbiotic formulation.

*Bacteroides uniformis* appears to be transitioning from mechanistic studies in animals toward clinical validation. This strain has been shown to suppress Th17 differentiation through α-MCA/HDCA/isoLCA, while also protecting against GPX4-mediated ferroptosis through alleviation of endoplasmic reticulum stress [69]. Preliminary clinical relevance data have been accumulated: *B. uniformis* tended to be enriched in long-term responders to vedolizumab, an intestinal-selective integrin inhibitor, and UC patients with higher baseline *Bacteroides* abundance appeared to respond better to a Mediterranean diet [14,15]. A current bottleneck may be determining whether the two mechanisms act in concert in humans. Given that the strength of the ferroptosis pathway may be related to dietary conditions, the answer to this question may help to judge whether dietary intervention could be applicable to specific patient populations.

*Clavispora lusitaniae* represents a translational path for fungal probiotics. This strain is the only fungal strain among the four that has reached Level 5 validation, and has been shown to alleviate colitis through production of IET via the tryptophan–AhR pathway [52]. The development of fungal biotherapeutics may have its own specific considerations: the activation pattern of host immunity by cell wall components, such as β-glucan, may differ from that of bacteria [100,101], and both colonization capacity and immunogenicity would need to be assessed de novo in humans. Therefore, the current translational tasks for this strain appear to be focused on basic safety evaluation and optimization of colonization ability, which may represent essential steps along the path toward clinical application of fungal probiotics.

*Lactobacillus johnsonii* presents a translational path that appears to rely on cross-feeding. This strain produces ILA and activates AhR to alleviate colitis, but its growth appears to depend on nicotinamide generated by *B. uniformis* through degradation of β-glucan. β-glucan intervention has been shown to increase the abundances of both *L. johnsonii* and *B. uniformis*, as well as ILA levels, in healthy individuals [53]. This ecological dependency suggests that the translational direction for this strain may not be a single-strain product, but rather a co-developed synthetic consortium with *B. uniformis*, or a component of β-glucan-adjuvant intervention.

Taken together, although these four candidate strains follow different paths and are at different stages, they appear to share a common direction: moving causally validated chains established in animal models progressively toward clinical application, through metabolite validation and target activation assessment in humans. The complete translational path of *A. muciniphila* from mechanistic validation to Phase II trials in metabolic diseases [108] may serve as a reference roadmap for the other candidates.

### 6.2. Postbiotics: Outer Membrane Vesicles as a Natural Metabolite Delivery Platform

Currently, live bacterial therapeutics face challenges including low colonization efficiency, poor strain consistency, and substantial inter-individual variability in responses. These limitations have prompted researchers to explore alternative delivery forms that do not rely on live bacterial colonization. Bacterial outer membrane vesicles (OMVs) are nanosized (20–400 nm) spherical lipid bilayer structures naturally secreted by Gram-negative bacteria during growth, and they carry a variety of bioactive molecules such as proteins, lipids, nucleic acids, and metabolites [109]. As key mediators of bacterium–host interactions, OMVs can cross the intestinal epithelial barrier and directly interact with intestinal epithelial cells and mucosal immune cells [110]. In essence, OMVs may serve as a natural vehicle for metabolite delivery, with the potential to deliver Level-5-validated effector metabolites in encapsulated form to host targets.

Current evidence suggests that the protective effects of OMVs in IBD are mainly exerted at three targets, with a clear gradient in the strength of evidence. The immunomodulatory direction has the most substantial support. OMVs derived from *Alistipes timonensis* have been shown to deliver SoLs into the host circulatory system, thereby inhibiting colitis [78]. This example directly links OMV-delivered metabolites to the Level-5 validation described earlier, representing the most complete causal chain in this area to date. In addition, OMVs from *Bacteroides thetaiotaomicron* can induce IL-10 production in healthy human dendritic cells, but this response appears to be markedly attenuated in IBD patients [111]. OMVs from *Bacteroides fragilis* have been reported to target PD-L1 through delivery of miR-5119, reducing neutrophil extracellular trap formation [86]. These findings indicate that OMVs can effectively modulate intestinal immunity, although their efficacy may be influenced by host immune status. Future studies may need to evaluate the differential effects of OMVs across patient subgroups to define the appropriate target populations.

Evidence for barrier protection is also relatively abundant. Extracellular vesicles from *Akkermansia muciniphila* and *Lactobacillus fermentum* have both been shown to upregulate tight junction protein expression and reduce intestinal permeability [112,113]. However, these effects appear to be highly similar to the known protective mechanisms of metabolites such as butyrate described earlier. At present, no study has clearly demonstrated that OMVs achieve barrier protection through delivery of a specific metabolite.

Evidence for microbiota remodeling is currently the least abundant. Extracellular vesicles from *Bifidobacterium longum* have been reported to retain some anti-inflammatory effects in pseudo-germ-free mice, suggesting that their actions may involve both microbiota-dependent and microbiota-independent pathways [114]. However, it remains difficult to distinguish whether the effects of OMVs on microbial composition are direct or indirectly mediated through host immune feedback. Distinguishing between these two possibilities may be a key question for future research on OMV-mediated microbiota remodeling.

**Figure 3:**
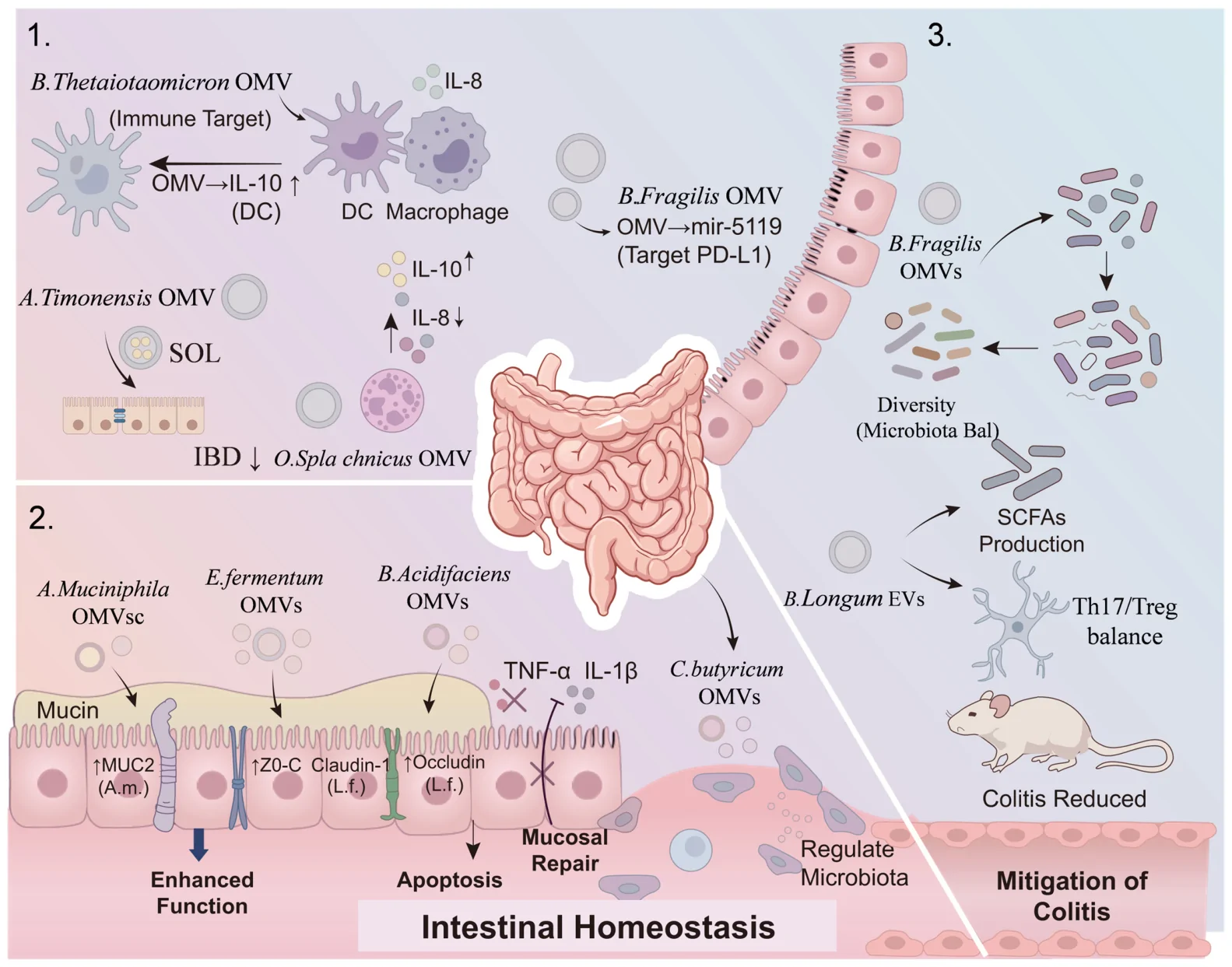
Protective mechanisms of gut microbiota-derived OMVs in IBD. The scheme highlights three major action axes (numbered 1–3): (1) targeting immune cells, (2) targeting the intestinal epithelial barrier, and (3) targeting gut microbiota remodeling. Arrows denote vesicle-mediated signaling pathways. OMVs may act as metabolite shuttles, delivering effectors to the host independently of live bacterial presence. In this figure, arrows “→” denote activation or positive regulation, while “↑” and “↓” indicate upregulation and downregulation of downstream molecules, respectively.

In summary, OMVs have shown protective potential at three levels—immune modulation, barrier protection, and microbiota remodeling (Figure 3). Nevertheless, at present, the effects of OMVs appear to be highly dependent on the natural products of specific strains, and the technical foundation for OMVs as a universal delivery platform has not yet been established. Whether OMVs can be upgraded from strain-specific natural products to a universal delivery platform remains an open question. Addressing this question may help to advance the field toward clinical translation.

### 6.3. Engineered Bacteria and Genetic Modification

The live bacterial therapeutics and postbiotics discussed above represent strategies of directly administering strains or their products, respectively, each with limitations in colonization efficiency or delivery precision. Engineered bacteria offer an alternative path: through genetic modification, validated metabolite synthesis pathways can be transferred into chassis strains with strong colonization capacity and suitability for industrial production, thereby enabling controlled release of effector molecules [115].

Let us take the IET pathway of *Clavispora lusitaniae P4013B* as an example [52]. This fungus naturally carries the Ehrlich pathway for IET synthesis, but its fermentation efficiency appears to be relatively low. The researchers identified two pyruvate decarboxylase genes, *pdc_2062* and *pdc_5239*, with higher catalytic efficiency, and transferred them into *Escherichia coli BL21* to construct two engineered strains. In mouse models of colitis, these two engineered strains were shown to continuously release IET locally in the gut, activate AhR signaling, downregulate lipid peroxidation, and enhance tight junction protein expression, thereby alleviating inflammation. The value of this strategy may lie in bypassing the weak colonization ability and low fermentation efficiency of the original fungus, and delivering an effector molecule already established through causal validation to a controllable and industrially feasible chassis strain for execution.

A similar logic may apply to other causally validated metabolites. For example, transferring the ILA synthesis pathway into a Lactobacillus chassis could also exert protective effects through the AhR pathway [116]; transferring antioxidant enzyme genes such as MnSOD into strains with strong gut colonization capacity could enable them to directly scavenge free radicals at intestinal mucosal lesions and inhibit ferroptosis [117]. These directions have received preliminary validation in animal models, but currently remain at the preclinical stage and have not yet entered clinical trials in IBD patients.

At present, the engineered bacteria strategy appears to share some common challenges with live bacterial therapeutics, including colonization efficiency, batch-to-batch consistency, and strain uniformity. However, there are also unique technical difficulties: stable maintenance of exogenous genes, risk of horizontal gene transfer, and more stringent safety regulatory requirements. In addition, whether the metabolic output of engineered bacteria can be stably maintained over the long term in the complex gut microbial environment remains to be further evaluated.

### 6.4. Clinical Translation: Challenges and Potential Solutions

Despite the promising preclinical evidence discussed above, translating causally validated strains and metabolites into effective clinical therapies faces substantial challenges that warrant a realistic appraisal.

A key biological barrier is the inflammatory milieu itself. Intestinal inflammation appears to limit the efficacy of live microbial therapeutics [118]. For example, *Escherichia coli* Nissle 1917 has shown efficacy in maintaining remission in ulcerative colitis, yet its benefit in treating active disease has not been clearly demonstrated [119,120]. This observation suggests that protective mechanisms identified under healthy or quiescent conditions may not be sufficient to overcome established inflammation.

Beyond this biological barrier, recent clinical setbacks further illustrate the translational gap. A rationally designed 16-strain consortium, VE202, failed to meet its primary endpoint in a Phase 2 trial for ulcerative colitis (NCT05370885). Such cases highlight the difficulty of translating even well-characterized consortia from preclinical models to human patients, and point to the need for better patient stratification and more robust predictive biomarkers.

In addition to these biological and clinical challenges, practical and regulatory hurdles also remain. Manufacturing consistency, batch-to-batch reproducibility, long-term stability, and effective delivery to target sites in the gastrointestinal tract are non-trivial technical challenges for live biotherapeutic products. Safety concerns, such as the risk of bacteremia in immunocompromised patients and the potential for horizontal gene transfer, also require careful evaluation [121]. Furthermore, the lack of standardized regulatory frameworks for potency assessment and quality control further complicates the path to clinical approval.

Beyond the specific challenges facing live biotherapeutic products, several broader methodological limitations of current causal microbiome research deserve acknowledgment, as they indirectly affect the translatability of preclinical findings. The field remains heavily dependent on animal models, which may not faithfully recapitulate human IBD pathophysiology. Strain-specific effects complicate generalization across different isolates of the same species, while dietary and pharmacological confounders are difficult to control and may influence both microbial composition and metabolite profiles. Substantial inter-individual variability in microbiome composition poses further challenges for reproducibility, and the bioavailability of metabolites following exogenous supplementation may differ considerably from that achieved through in situ microbial production. Moreover, the lack of standardized metabolomic methodologies hampers cross-study comparison. These issues collectively represent major barriers to clinical translation and underscore the need for more standardized and human-relevant experimental approaches.

To move beyond these obstacles, several strategies have been proposed. One approach is patient stratification based on baseline microbiota composition or inflammatory status. Retrospective analyses have suggested that pre-treatment microbial features may predict clinical outcomes [122,123]. In addition to microbial features, host inflammatory markers have also been explored as guides for treatment selection. For instance, the Treat-smart study demonstrated that IBD patients with a TNF-α-dominant inflammatory pathway achieved higher remission rates with anti-TNF-α therapy, supporting the feasibility of pathway-based patient stratification [124]. Another possibility is combination therapy, pairing live bacterial therapeutics with anti-inflammatory agents to create a more permissive environment for colonization and metabolic activity [125,126]. For live products, an alternative direction is the use of postbiotics or purified metabolites instead of live bacteria. This strategy could bypass some safety and manufacturing concerns while retaining effector functions validated at Level 5 [127]. Improved delivery systems, including encapsulation or biofilm-based formulations, may also enhance survival and release of therapeutics at target sites [128]. Efforts to address the broader methodological limitations—such as developing standardized metabolomic protocols and improving cross-species translatability—will also be critical, though these require longer-term investment at the field level.

In summary, while the causal evidence pipeline has advanced considerably, clinical translation remains a challenging step. Acknowledging these limitations and actively exploring potential solutions will be important for moving the field toward tangible patient benefit.

## 7. Conclusions and Perspectives

Gut microbiota research is progressively shifting from correlative description toward causal mechanistic validation. In this review, we constructed a five-level progressive evidence framework spanning from multi-omics association discovery to molecular causal validation. Based on this framework, we systematically synthesized the single-bacterium–metabolite–host target axes across four pathways—AhR/tryptophan metabolism, bile acid/nuclear receptor signaling, short-chain fatty acid/vitamin/energy metabolism, and cell death-related pathogenic mechanisms. A total of more than 30 axes with evidence at Level 4 or above were included, most of which focus on conservation mechanisms.

This evidence map reveals several structural features. The marked imbalance between protective and pathogenic causal evidence partly reflects the inherent limitation of widely used models such as DSS and TNBS, which favor resolution over initiation of inflammation. This bias is not entirely coincidental. Acute chemical injury models offer relatively standardized protocols, short experimental cycles, and high throughput, making them well suited for testing whether interventions promote tissue repair. Consequently, they have been extensively adopted. In contrast, investigating pathogenic mechanisms often requires spontaneous inflammation models, such as IL-10 knockout mice or T-bet^−/−^ × Rag2^−/−^ mice, which are technically demanding, time-consuming, and less amenable to high-throughput screening. These practical barriers have, to some extent, shifted the research focus toward protective mechanisms.

To help rebalance this landscape, future studies could adopt a tiered validation pipeline—using patient-derived intestinal organoid co-culture systems for initial screening of pathogenic candidates, followed by validation in spontaneous colitis models to assess their roles in disease initiation and persistence. This complementary strategy may provide a more complete understanding of host–microbe interactions in IBD.

In addition, multiple pathways appear to share downstream effector nodes, and the same strain may act through several metabolite axes simultaneously, suggesting that single-metabolite supplementation strategies might face dilution of efficacy due to network redundancy. Of particular note, all Level 5 evidence currently originates from animal models, and direct in vivo data on actual metabolite exposure levels and target engagement efficiency in the human gut remain lacking. It should be acknowledged that while these animal models—particularly germ-free and gnotobiotic mice—have provided invaluable mechanistic insights, their direct translatability to human IBD is not guaranteed. The inflammatory microenvironment, genetic background, and microbial ecology in humans differ substantially from those in controlled animal settings. Consequently, causal relationships established in animal models may not fully recapitulate the complex pathophysiology of human IBD. Future studies should prioritize prospective validation of the identified strain–metabolite–host axes in well-characterized patient cohorts, and incorporate human organoid-based screening or pharmacokinetic assessments to better predict whether the effector molecules can reach therapeutic concentrations at target sites in the human gut. Beyond the intestinal tract, the gut–liver axis has also emerged as a relevant dimension in IBD, as gut microbiota dysbiosis is increasingly linked to hepatobiliary complications through shared inflammatory and metabolic pathways [129]. Mesenchymal stem cells and their exosomes have been explored as modulators of the gut microbiota in both liver diseases and IBD [130], though these remain at an early stage of investigation.

Another notable gap in the current evidence landscape concerns the viral component of the gut microbiota. Although phages and eukaryotic viruses are increasingly recognized as contributors to IBD pathogenesis through direct immune modulation and indirect effects on bacterial composition [131], our understanding of their precise causal roles remains limited. To date, causal validation of individual phages or eukaryotic viruses in the IBD context remains scarce, which may be attributed in part to the absence of standardized colonization models for viruses, the limited availability of molecular tools to establish virus–host causality, and the inherent technical challenges in viral sequencing and functional annotation. Given that phages can influence bacterial functional output and that eukaryotic viruses may independently shape inflammatory phenotypes, further investigation into viral contributions represents a valuable direction for future research.

These gaps collectively point toward priority directions for future work. At the methodological level, we suggest expanding causal validation from protective toward pathogenic mechanisms, with greater use of spontaneous inflammation models, such as IL-10 knockout, T-bet^−^/^−^ × Rag2^−^/^−^ mice, and patient-derived organoid co-culture systems. In parallel, the development of tools and models for studying phage host and eukaryotic virus host interactions merits further consideration, as this area has yet to be systematically explored in the context of causal validation. A tiered pipeline, beginning with organoid based screening, followed by confirmation in animal models, and ultimately progressing to human assessment, could facilitate higher-throughput causal validation for both bacterial and viral candidates.

At the translational level, all candidate strains, before entering IBD clinical trials, would need to address a core baseline question: whether the target metabolite is stably produced in the human gut at concentrations sufficient to reach therapeutic thresholds. From a field perspective, the single-bacterium–metabolite axis has established reference translational paths in other disease areas. Akkermansia muciniphila has completed the full translational chain in overweight/obese and type 2 diabetic patients, from mechanistic validation through Phase I safety assessment to Phase II efficacy exploration, achieving quantifiable improvements in metabolic endpoints [108]. In Parkinson’s disease, randomized double-blind trials of short-chain fatty acid supplementation have also demonstrated clinically meaningful endpoint benefits [132]. These examples from metabolic and neurodegenerative diseases may offer methodological templates for IBD translational research. IBD research could consider incorporating a human pharmacokinetic pre-evaluation step before initiating randomized controlled trials of metabolites or live bacterial products, using human intestinal organoid models, gut perfusion systems, and stable isotope tracing to determine the exposure levels, target occupancy, and dose–response relationships of candidate metabolites in the human gut. However, it should be noted that these human assessment techniques generally have limitations in throughput, high detection costs, and in some cases ethical constraints associated with invasive procedures, making large-scale cohort application challenging in the near term. Causal chains that lack human dose–exposure–effect data may not yet be sufficiently mature for clinical translation.

At the clinical decision-making level, given that the current metabolomics-based predictive AUC for biologic response is only 0.70, future studies could move beyond the conventional approach of screening differential metabolites and toward constructing composite biomarkers that reflect causal axis activity. For example, integrated assessment of fecal total AhR ligand capacity, secondary bile acid profiles, and microbial butyrate-producing capacity could be incorporated into companion diagnostic models for anti-TNF or anti-IL-23 therapies.

Substantive progress in these key directions may represent a core prerequisite for moving IBD microbiome research from basic mechanisms toward clinical application. The causal evidence map and the five-level grading framework proposed in this review are intended to provide a reference standard for evidence grading and directional guidance for translational research in the field.

## Figures and Tables

**Table 1:** Summary of causal evidence for the AhR/tryptophan metabolism and bile acid/nuclear receptor signaling pathways.

Classification	Strains	Effector Metabolites	Host Target/ Receptor	Signal Pathway/ Mechanism	Direction of Effect	Validation of the Model	Evidence Level	References
AhR/ tryptophan metabolic axis	*Peptostreptococcus* spp.	Indoleacrylic acid (IA)	Nrf2	Nrf2 activation → MUC2 increase → barrier protection	Protect	DSS mice, IA supplementation	Level 5	[48]
	*Lactobacillus reuteri*	Indole-3-acetic acid (IAA)	AhR	AhR → vimentin sulfation	Protect	DSS mice metabolite supplementation, iaaM knockout	Level 5	[49]
	*Clavispora lusitaniae P4013B*	Indole-3-ethanol (IET)	AhR	AhR → CYP1A1 ↑	Protect	Antibiotic depletion, GF single-bacterial colonization, IET supplementation, AhR gene knockout mice, engineered bacteria	Level 5	[52]
	*Lactobacillus johnsonii* (promoted by cross-feeding with *Bacteroides uniformis*)	Indole-3-lactic acid (ILA)	AhR	AhR → IL-22 → Muc2 ↑	Protect	Multimodal analysis, antibiotic depletion, fecal microbiota transplantation (FMT), single-bacterial colonization, ILA/NAM reconstitution, AhR antagonists	Level 5	[53]
	*Campylobacter concisus*	Indole metabolites (mainly trisindoline)	AhR	AhR → proinflammatory cytokines ↑	Pathopoiesia	Non-targeted metabolomics, trisindoline taken orally directly	Level 5	[51]
	*Lactobacillus vaginalis* *BNCC186140*	3-Iodoindoleacetic acid (IAA)	PPAR signaling pathway	PPAR → Th17/Treg balance	Protect	Multimodal analysis, antibiotic depletion, FMT, single-bacterial colonization, IAA supplementation	Level 5	[54]
	*Akkermansia muciniphila*	Indole-3-ethanol (IET)	AhR	AhR → IL-22 → barrier repair	Protect	Multimodal analysis, antibiotic depletion, FMT, I3E supplementation	Level 4	[55]
	*Porphyromonas gingivalis*	Linoleic acid (LA)	AhR	AhR → Stat1 → Th17/Treg unbalance	Pathopoiesia	Multimodal analysis, single-bacterial gavage, LA supplementation	Level 5	[56]
	*Lactobacillus reuteri D8*	Indole-3-aldehyde (IAld)	AhR	AhR → IL-22 → STAT3 → ISC proliferation	Protect	Single-bacterial colonization, IAld supplementation, AhR inhibitors	Level 5	[57]
Bile acid/ nuclear receptor signaling axis	*Akkermansia muciniphila*	p-HPAA, DCA, UDCA	FXR	Regulating bile acid metabolism → FXR-dependent inhibition of XBP1u → protecting the barrier	Protect	16S rRNA, FMT, FXR knockout mice; single-bacterial gavage;	Level 5	[58]
	*Candida metapsilosis* *M2006B*	Unicyclic sesquiterpenoids (F4, F5)	FXR	Activating FXR → upregulating SHP and FGF15 → inhibiting inflammation	Protect	Antibiotic depletion, GF single bacteria, FXR knockout mice, metabolite supplementation	Level 5	[59]
	*Odoribacter splanchnicus*	Lithocholic Acid (LCA)	NLRP3/GSDMD	Inhibition of NLRP3-GSDMD → reduction in NET formation	Protect	16S rRNA, antibiotic depletion, FMT, single bacteria, LCA supplementation, gene knockout	Level 5	[60]

In the Signal pathway/mechanism column, arrows indicate regulatory relationships: “↑” indicates upregulation/increased expression of the downstream molecule.

**Table 2:** Summary of experimentally validated causal evidence for the short-chain fatty acid/vitamin/energy metabolism and cell death/pathogenic mechanisms.

Classification	Strains	Effector Metabolites	Host Target/ Receptor	Signal Pathway/Mechanism	Direction of Effect	Validate the Model	Evidence Level	References
Short-chain fatty acid/ vitamin/ energy metabolism	*Faecalibacterium prausnitzii*	Butyric acid	HDAC	Inhibition of HDAC → increase in Foxp3^+^ Tregs → balance of Th17/Tregs	Protect	Single-bacterial colonization of GF mice	Level 4	[76]
	*Akkermansa muciniphila*	Spermidine (SPD)	PI3K/AKT/NF-κB pathway	Inhibition of PI3K/AKT/NF-κB phosphorylation → downregulation of inflammatory factors	Protect	Antibiotic depletion, FMT, SPD replenishment	Level 5	[77]
	*Alistipes timonensis*	Sulfanilamide B (SoL B)	PI3K/AKT/NF-κB pathway	Inhibit LPS-induced IL-1β, IL-6, and TNF-α; regulate lipid profile	Protect	Multimodal analysis, IL-10-deficient mice (single-bacterial colonization + purified SoL B intraperitoneal injection)	Level 5	[78]
	*Lactobacillus intestinalis*	Retinoic acid (RA, including aRA and 13cisRA)	RAR/RXR	Convert dietary vitamin A into active retinoic acid	Protect	Multimodal analysis, single-bacterial colonization after antibiotic treatment of mice, and colonization in GF mice	Level 4	[79]
	*Bifidobacterium pseudolongum* *ATCC25526*	R-equol	Estrogen receptor	Activate ER → upregulate Foxp3 → increase Treg	Protect	Multimodal analysis, antibiotic depletion, single-bacterial colonization, metabolite replenishment	Level 5	[71]
	*Lactobacillus reuteri* *BNCC192190*	α-ketoglutaric acid	/	Promote bacterial growth → activate Wnt/β-catenin → repair the barrier	Protect	16S rRNA sequencing, targeted energy metabolism profiling, single-bacterial colonization	Level 4	[80]
	*Akkermansia muciniphila*	N-acetylspermidine	PIM1, HDAC2, C1GALT1C1	Upregulation of HDAC2 → reduction in chromatin openness at the TP73 site → promotion of α1,2-galactosylation	Protect	Multimodal analysis, single bacteria, metabolite supplementation	Level 5	[72]
	*Lacticaseibacillus rhamnosus GG*	Methylnicotinamide (MNA)	/	Promote the metabolism of tryptophan and nicotinamide → upregulate RNF43 → promote epithelial differentiation	Protect	Multimodal analysis, single-bacterial colonization in GF mice, MNA direct supplementation	Level 5	[73]
	*Bacteroides fragilis*	Short-chain fatty acids (SCFAs), especially butyrate	NLRP3 inflammasome	Negative regulation of NLRP3 → inhibition of IL-18/IL-1β → restriction of CAC	Protect	Database analysis, clinical fecal qPCR, antibiotics depletion, single-bacterial colonization	Level 4	[81]
	*Bacillus velezensis PGM541*	Butyric acid (BA)	FFAR2 (Free Fatty Acid Receptor 2)	Activation of FFAR2 → promotion of intestinal epithelial cell proliferation	Protect	Intestinal organoid screening, single-bacterial colonization, butyric acid supplementation, antagonists	Level 5	[74]
	*Limosilactobacillus reuteri* *DS0384*	N-formylglutamic acid (NCG)	/	Promote intestinal epithelial maturation, increase the proliferation of intestinal stem cells, protect from cytokine-induced intestinal epithelial damage, and repair the barrier	Protect	Single-bacterial colonization, human intestinal organoids (hIOs) model; intestinal stem cell spheroid model, direct supplementation with NCG	Level 5	[75]
Cell death and pathogenic mechanisms	*Ruminococcus gnavus*	Galactomannan	TLR4	TLR4 → dendritic cells → TNF-α/IL-8 ↑	Pathopoiesia	DC cell culture, sterile mouse colonization	Level 4	[47]
	*Bacteroides uniformis*	/	GPX4, HSPA5, ATF4	Inhibit endoplasmic reticulum stress → protect GPX4-mediated ferroptosis	Protect	Single-bacterial colonization in GF mice	Level 4	[69]
	*Lactobacillus gasseri*	/	GPX4/GSH axis	Activating the GSH/GPX4 pathway → inhibiting ferroptosis	Protect	Multimodal analysis, single-bacterial colonization	Level 4	[82]
	*Enterococcus faecalis*	Host protein FABP2	EF3041	Hijack FABP2 → activate quorum sensing → excessive proliferation	Pathopoiesia	Antibiotic depletion, single-bacterial colonization	Level 4	[83]
	*Parasutterella excrementihominis*	Succinic acid (Suc), 6-hydroxyhexanoic acid (6-HHA)	SUCNR1, GPR84	Activation of NLRP3/GSDMD → induction of NETosis	Pathopoiesia	Antibiotic depletion, FMT, single-bacterial colonization, metabolite supplementation, gene knockout	Level 5	[84]
	*Muribaculaceae*	Melatonin	MT2 receptor	Inhibition of TRAF6/p65-NFATc1 → anti-apoptosis	Protect	16S rRNA, metabolome, FMT, melatonin supplementation, MT2 antagonist	Level 5	[85]

In the Signal pathway/mechanism column, arrows indicate regulatory relationships: “↑” indicates upregulation/increased expression of the downstream molecule.

## Data Availability

No new data were created or analyzed in this study. Data sharing is not applicable to this article.
